# Relationship between the sFlt-1/PlGF ratio and the optical coherence tomographic features of chorioretina in patients with preeclampsia

**DOI:** 10.1371/journal.pone.0261287

**Published:** 2021-12-09

**Authors:** Jaekyoung Lee, Jin Gon Bae, Yu Cheol Kim

**Affiliations:** 1 Department of Ophthalmology, Keimyung University School of Medicine, Daegu, Korea; 2 Department of Obstetrics, Keimyung University School of Medicine, Daegu, Korea; University of Florida, UNITED STATES

## Abstract

This study aimed to evaluate the correlation between ophthalmologic factors and the serologic indicator soluble fms-like tyrosine kinase 1 (sFlt-1): placental growth factor (PlGF) ratio in patients with preeclampsia using optical coherence tomography (OCT) and OCT angiography (OCT-A). A total of 52 pregnant patients (104 eyes) diagnosed with preeclampsia were recruited during their hospital stay. The associations between the sFlt-1/PlGF ratio and chorioretinal measurements, including the choroidal thickness (CT), foveal avascular zone, vascular density, and ganglion cell layer+ were evaluated. Central and nasal subfield CT of the left eye (p = 0.039; p = 0.010) and nasal subfield CT of the right eye (p = 0.042) were lower in the high sFlt-1/PlGF ratio group (≥38). Pearson’s correlation test showed a negative correlation between the sFlt-1/PlGF ratio and central subfield CT; however, this was not statistically significant (p = 0.648). Linear regression analysis revealed a significant association between the sFlt-1/PlGF ratio and central subfield CT (β coefficient, -6.66; p = 0.01) and between sFlt-1 and central subfield CT (β coefficient, -5.65; p = 0.00). Thus, an increase in the sFlt-1/PlGF ratio resulted in a decrease in central subfield CT.

## Introduction

Preeclampsia refers to the new onset of hypertension and proteinuria after 20 weeks of gestation, and is a leading cause of prenatal and maternal mortality and morbidity, affecting 2–5% of pregnancies [1,2]. Although the pathophysiology of preeclampsia is not fully understood, this condition is associated with abnormal placental perfusion resulting from the inadequate remodeling of maternal spiral arteries, causing increased systemic vascular resistance and endothelial dysfunction [3,4]. Approximately 40% of patients with preeclampsia complain of subjective visual symptoms, including decreased vision, visual field defects, and numerous ocular changes, including increased central corneal thickness and corneal curvature, decreased corneal sensitivity, and intraocular pressure [5–7]. Changes in blood-ocular barrier, choroidal thickness (CT), and circulation in preeclampsia occur [8–10]. Additionally, in preeclampsia, circulating maternal anti-angiogenic factors, such as soluble fms-like tyrosine kinase 1 (sFlt-1), are increased, and placental growth factor (PlGF) levels are decreased. A high sFlt-1/PlGF ratio has recently been found to be associated with vasoconstriction and endothelial damage in patients with preeclampsia; thus, influencing the ocular environment [11–13].

Swept-source optical coherence tomography (SS-OCT) can visualize the choroid structure at a higher resolution and measure the CT [14,15]. Moreover, OCT angiography (OCT-A) can provide detailed imaging of the vascular structures of the retina and choroid repeatedly and noninvasively. Using this OCT technology, researchers have studied the correlation between maternal serologic changes in preeclampsia and ocular characteristics [16,17]. However, to our knowledge, no research on the relationship between the sFlt-1/PlGF ratio and ocular structural changes has been reported to date. This study aimed to elucidate the correlation between the sFlt-1/PlGF ratio and chorioretinal microvascular changes using OCT and OCT-A in patients with preeclampsia.

## Materials and methods

### Study population

This study was designed as a retrospective, comparative case series conducted in a single hospital. The study was performed in accordance with the Declaration of Helsinki and was approved by the Institutional Review Board of the Keimyung University Hospital (approval number 2018-06-002). Informed consent was waived because of the retrospective nature of the study. The data were anonymized and aggregated before access and analysis. We included 104 eyes of 52 pregnant women diagnosed with preeclampsia referred to our Ophthalmology Department between November 2019 and October 2020. Preeclampsia was defined according to the criteria of the American College of Obstetricians and Gynecologists [18]. The diagnostic criteria were a new onset of hypertension (systolic blood pressure (SBP) ≥140 mmHg, diastolic blood pressure (DBP) ≥90 mmHg, or both) and proteinuria after 20 weeks of gestation. The exclusion criteria were as follows: previous ocular surgery, any ocular pathology affecting best-corrected visual acuity, and inability to undergo obstetrical and ophthalmologic evaluation.

### Clinical data collection

At the initial visit, the medical history of each participant was taken to assess their demographic and obstetrical characteristics. On admission and ophthalmic examination, systolic and diastolic blood pressure (BP) were measured using an automated blood pressure machine. All patients were allowed to rest for at least 5 min before measurements for stable hemodynamic conditions. The body mass index (BMI, kg/m^2^) was calculated. Serum levels of sFlt-1 and PlGF were examined, and the sFlt-1/PlGF ratio was obtained. Routine serum and urine analyses were performed, including values considered significant in preeclampsia, such as urine and serum protein, creatinine, and albumin concentrations. Furthermore, the use of intravenous magnesium and blood pressure-lowering agents, such as oral nifedipine and intravenous hydralazine, was investigated.

All patients underwent comprehensive ophthalmic assessment, including best-corrected visual acuity and intraocular pressure using Goldmann applanation tonometry (AT 900®, Haag-Streit, Koniz, Germany), spherical equivalent, and slit-lamp examination of the anterior and posterior segments.

**Fig 1:**
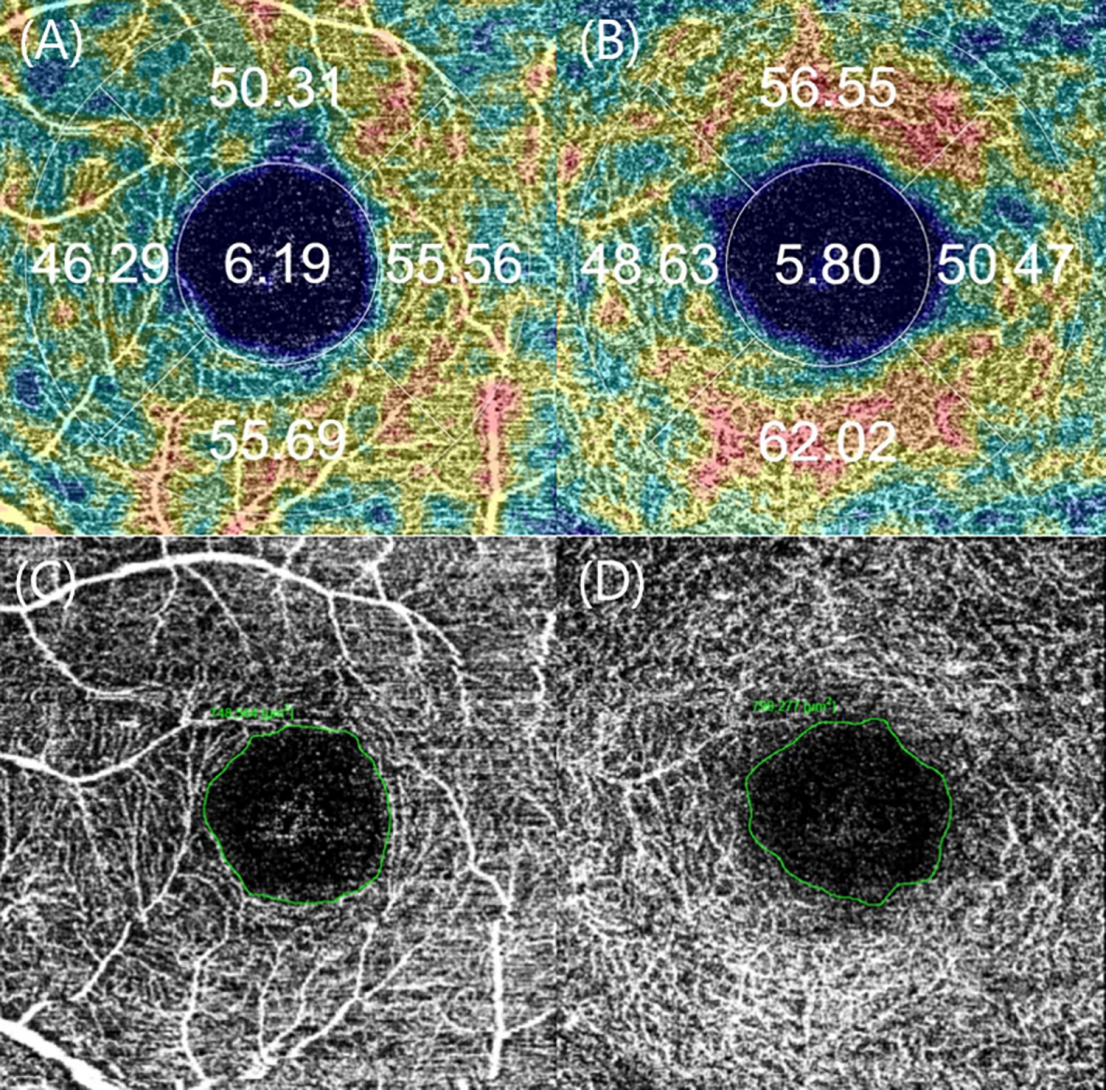
Macular en face images of an optical coherence tomographic angiography scan. The vascular densities of superficial (A) and deep (B) capillary complexes are automatically calculated. The foveal avascular zone (FAZ) borderline is analyzed manually and the FAZ areas of the superficial (C) and deep (D) capillary complexes are calculated automatically using the area measurement tool in IMAGEnet 6.

Images of OCT and OCT-A were acquired using SS-OCT (DRI OCT Triton®, Topcon, Tokyo, Japan) by a skilled examiner in the afternoon to avoid diurnal variations of the choroid. The average thickness of the choroid and ganglion cell layer+ (GCL+) in the Early Treatment Diabetic Retinopathy Study (ETDRS) grid was automatically measured using the thickness map of the macula from the raster scan. In OCT-A, the 3.0×3.0 mm macular en face images were divided into superficial capillary plexus (SCP) and deep capillary plexus (DCP) automatically. Vessel density (VD), defined as the ratio of the area of the vessel and microvasculature to the total area, was automatically measured from the SCP and DCP images of IMAGEnet 6 (version 1.22, Topcon, Tokyo, Japan). Among the ETDRS subfields, the central area was defined as a centered 1.0-mm-diameter circle; the annular area that remained after subtraction of the central area from the 3.0-mm-diameter circle was defined as the paracentral area. Paracentral areas were divided into four areas: temporal, nasal, superior, and inferior. The superficial and deep foveal avascular zone (FAZ) borderline was analyzed by a retinal specialist using IMAGEnet 6 (Fig 1).

### Statistical analysis

Data were calculated as mean ± standard deviation (SD). A total of 52 study participants (104 eyes) were divided into two groups according to the sFlt-1/PlGF ratio value 38, based on the study by Zeisler et al.: group 1 (<38) and group 2 (≥38) [12].

SPSS version 20.0, for Windows (IBM Co., Armonk, NY, USA), was used for statistical analyses. We used the independent Student’s t-test, Pearson’s test, and linear regression test. Independent Student’s t-test was used to evaluate the difference between the means of OCT and OCT-A parameters between the two groups. Pearson’s correlation test and the linear regression test were used to examine the correlation between the sFlt-1/PlGF ratio and variable factors, including CT. Statistical significance was set at p<0.05.

## Results

Twenty patients were included in group 1 and 32 patients in group 2. The average sFlt-1/PlGF ratio of group 1 was 11.53±8.51 and that of group 2 was 398.77±387.15. Systolic and diastolic BP and fetal growth retardation were significantly higher in group 2 (p = 0.008, 0.025, and <0.001, respectively) compared to group 1. Intravenous magnesium (Magunesin®, Daewon, Seoul, Korea) was administered to eight patients in group 2 to prevent eclampsia (p = 0.042) (Table 1).

**Table 1:** Demographical and clinical features of pregnant women with preeclampsia.

	Group 1 (n = 20)	Group 2 (n = 32)	*p*-value*
Age (years)	32.50 ± 4.65	34.88 ± 3.90	0.053
Right eye BCVA (LogMAR)	0.33 ± 0.33	0.23 ± 0.32	0.332
Left eye BCVA (LogMAR)	0.26 ± 0.31	0.21 ± 0.30	0.554
Right eye IOP (mmHg)	15.50 ± 3.07	14.09 ± 3.06	0.114
Left eye IOP (mmHg)	15.65 ± 2.66	13.72 ± 3.62	**0.045**
Right eye SE (D)	-1.66 ± 1.67	-1.66 ± 1.63	1.000
Left eye SE (D)	-1.23 ± 1.77	-1.39 ± 1.65	0.743
Body mass index (kg/m^2^)	30.56 ± 5.85	28.91 ± 5.94	0.332
Gestational age at delivery (weeks)	32.31 ± 11.25	32.82 ± 3.12	0.844
PMA (weeks)	29.53 ± 4.54	31.02 ± 3.57	0.195
Initial SBP (mmHg)	143.50 ± 19.27	146.25 ± 26.82	0.692
Initial DBP (mmHg)	87.50 ± 13.33	87.19 ± 15.50	0.941
SBP on exam (mmHg)	159.50 ± 19.05	173.28 ± 16.39	**0.008**
DBP on exam (mmHg)	93.50 ± 13.87	101.72 ± 8.85	**0.025**
Magnesium use (%)	0.0	25.00	**0.042**
BP-lowering agent use (%)	70.00	87.50	0.232
Twins (%)	15.00	15.62	1.000
Fetal growth retardation (%)	5.00	65.62	**<0.001**
sFlt-1/PlGF ratio	11.53 ± 8.51	398.77 ± 387.15	**<0.001**

*The p-value was calculated using an independent t-test or Pearson’s chi-square test.

BCVA, best-corrected visual acuity; IOP, intraocular pressure; SBP, systolic blood pressure; DBP, diastolic blood pressure; SE, spherical equivalent; PMA, postmenstrual age; OD, oculus dexter; OS, oculus sinister; D, dioptre; sFlt, soluble fms-like tyrosine kinase 1; PlGF, placental growth factor; BP, blood pressure.

The CT of the nasal area of the right eye and those of the central and nasal areas of the left eye were significantly lower in group 2 (p = 0.042, 0.039, and 0.010, respectively) than in group 1. VD and FAZ in the superficial and deep retina and the GCL+ showed no difference between the two groups (Table 2). The central subfield CT showed a positive correlation with peak SBP (r = 0.035, p = 0.805), and a negative correlation with peak DBP (r = -0.056, p = 0.695); however, both were not statistically significant.

**Table 2:** Choroidal and retinal vascularity parameters in pregnant women with preeclampsia.

		Group 1 (n = 20)	Group 2 (n = 32)	p-value*
OD	CT (C)	232.85 ± 53.89	209.91 ± 56.14	0.152
	CT (T)	221.50 ± 50.86	220.59 ± 49.15	0.949
	CT (N)	227.70 ± 50.35	196.69 ± 53.02	**0.042**
	CT (S)	230.05 ± 56.41	222.88 ± 47.17	0.623
	CT (I)	223.90 ± 57.70	214.66 ± 59.18	0.583
	GCL+ (C)	41.00 ± 6.84	37.31 ± 10.77	0.137
	Superficial VD (C)	21.08 ± 4.94	20.87 ± 6.46	0.901
	Deep VD (C)	17.10 ± 4.81	21.04 ± 13.39	0.138
	Superficial FAZ	338.95 ± 138.70	361.35 ± 108.62	0.527
	Deep FAZ	467.84 ± 334.88	488.71 ± 222.87	0.811
OS	CT (C)	229.85 ± 39.70	198.84 ± 57.38	**0.039**
	CT (T)	229.95 ± 37.74	213.16 ± 52.82	0.222
	CT (N)	224.65 ± 38.81	186.66 ± 55.21	**0.010**
	CT (S)	222.75 ± 47.87	205.06 ± 57.31	0.255
	CT (I)	234.15 ± 50.84	214.72 ± 49.29	0.178
	GCL+ (C)	40.70 ± 7.09	39.56 ± 7.84	0.600
	Superficial VD (C)	21.06 ± 4.12	21.08 ± 7.64	0.987
	Deep VD (C)	17.53 ± 4.84	19.52 ± 8.55	0.296
	Superficial FAZ	325.16 ± 166.31	374.00 ± 150.47	0.290
	Deep FAZ	486.95 ± 352.59	503.65 ± 220.72	0.854

*The p-value was calculated using an independent t-test.

CT, choroidal thickness; C, central; T, temporal; N, nasal; S, superior; I, inferior; GCL, ganglion cell layer; VD, vascular density; FAZ, foveal avascular zone; OD, oculus dexter; OS, oculus sinister.

The Pearson’s correlation test showed a negative correlation between the sFlt-1/PlGF ratio and central subfield CT; the association was not statistically significant (p = 0.648) (Table 3). However, the linear regression analysis revealed a significant association between the sFlt-1/PlGF ratio and central subfield CT (β coefficient, -6.66; p = 0.01), and between sFlt-1 and the central subfield CT (β coefficient, -5.65; p<0.001) (Table 4).

**Table 3:** Correlation between retinal factors and serologic indicators.

	sFLT-1/PlGF ratio		sFLT-1		PlGF	
	Coefficient (*r*)	p-value*	Coefficient (*r*)	p-value*	Coefficient (*r*)	p-value*
CT (C)	-0.065	0.648	-0.087	0.541	0.028	0.844
GCL+ (C)	-0.1868	0.185	-	-	-	-
Superficial VD (C)	0.0243	0.866	-	-	-	-
Deep VD (C)	0.2443	0.084	-	-	-	-
Superficial FAZ (C)	0.1125	0.437	-	-	-	-
Deep FAZ (C)	-0.0097	0.947	-	-	-	-

* The p values and correlation coefficients (*r*) were calculated using parametric correlation analysis.

sFlt-1, soluble fms-like tyrosine kinase 1; PlGF, placental growth factor; CT, choroidal thickness; GCL, ganglion cell layer; VD, vascular density; FAZ, foveal avascular zone; C, central.

**Table 4:** Choroidal thickness associated with sFLT-1/PlGF ratio and sFLT-1.

	β (95% confidence interval)		
	CT(C)		
	Parameter estimate (β)	Standard error	p-value*
sFlt-1/PlGF ratio	-6.66	2.70	**0.017**
sFlt-1	−5.65	1.34	**<0.001**

*The p-value was calculated using a linear regression test.

sFLT-1, soluble fms-like tyrosine kinase 1; PlGF, placental growth factor; CT, choroidal thickness; C, central.

## Discussion

Preeclampsia is initiated with an inadequate invasion of trophoblasts in early pregnancy, leading to placental ischemia, increasing the release of factors associated with maternal systemic endothelial dysfunction [3]. Systemic endothelial dysfunction, vasospasm, and vasoconstriction are caused by various factors, including higher vascular sensitivity to angiotensin II and decreased formation of vasodilators, such as nitric oxide. Consequently, cytotoxic, vasogenic edema, and multiorgan failure occur. Numerous ocular and vascular changes that occur in preeclampsia are well known, including retinal hemorrhage or edema, Elschnig spots, central serous chorioretinopathy, generalized constriction of retinal arterioles, and retinal detachments [19,20].

Several studies have been conducted on the relationship between the sFlt-1/PlGF ratio and the severity of preeclampsia. In normotensive pregnancy, the sFlt-1 levels are stable until the middle stage of gestation, and they increase steadily at 33–36 weeks, corresponding to the late decrease in PlGF levels [21]. sFlt-1 acts by adhering to the receptor-binding domains of PlGF and vascular endothelial growth factor (VEGF), preventing interaction with endothelial receptors on the cell surface [22]. Thus, placental vascular growth is tempered by an increase in anti-angiogenic sFlt-1 and a decrease in angiogenic VEGF and PlGF levels. However, in women with preeclampsia, sFlt-1 increases earlier in gestation and reaches a higher concentration than in normal pregnancy [22]. Therefore, in preeclampsia, the sFlt-1/PlGF ratio is increased sooner, indicating that the normal governing of placental growth is exaggerated and the anti-angiogenic “brakes” are applied too early.

The choroid primarily consists of vascular components with the highest blood flow per unit mass in the human body, controlled by the autonomous system. Accordingly, it is more sensitive to systemic vascular changes in preeclampsia compared with other organs. Valluri et al. reported choroidal ischemia and severe damage to the choroidal vasculature in preeclampsia using indocyanine green angiography [23]. In our study, the nasal side CT tends to show more difference between group 1 and 2. We suspect that if there is a change in the optic nerve head (ONH), the most affected and vulnerable part would be the nasal portion due to its proximity. Changes in the ONH might occur due to systemic changes, such as hypertension, which may explain our findings. However, we did not investigate the changes in the ONH separately, and no cases of morbid changes in the ONH have been reported to date; therefore, the definite correlation remains unknown. Moreover, in our study, no significant correlation was found between CT and systolic, diastolic BP. This finding is consistent with the previous report speculating that the chorioretinal pathology and hypertension are independent results of systemic imbalance, although systemic imbalance of vascular factors affects both BP and ocular vascular condition, and the CT is not directly associated with BP, but associated with protein-creatinine ratio (PCR) in preeclampsia [24].

The choroid is responsive to circulating angiogenic factors, such as VEGF, PlGF, and soluble endoglin, which are involved in the pathogenesis of preeclampsia [22,25]. PlGF is a member of the VEGF family. It is associated with pathological angiogenesis and inflammation in retinal vascular diseases [26]. High levels of PlGF have been found in the aqueous humor, vitreous, and retina of patients exhibiting retinopathies, including diabetic retinopathy and neovascular age-related macular degeneration. Stern-Ascher et al. reported a positive correlation between PlGF and central CT in pregnant women with severe preeclampsia, emphasizing the relationship between the degree of disease severity and the magnitude of choroidal thickening [16].

In this study, Pearson’s test showed a negative correlation between the sFlt-1/PlGF ratio and the central subfield CT; nevertheless, this was not statistically significant. However, the linear regression test revealed a significant decrease in central CT associated with an increase in the sFlt-1/PlGF ratio. This signifies that preeclampsia patients with higher sFlt-1/PlGF ratios may have a lower CT. In women with severe preeclampsia, sFlt-1, a circulating anti-angiogenic protein, increases, and conversely, PlGF, an angiogenic protein, decreases in early gestation. The augmented anti-angiogenic effect impacted the choroidal tissue with high VD, resulting in a decreased CT. Moreover, we speculate that as patients with high-risk preeclampsia with increased sFlt-1/PlGF ratio suffer more severe systemic endothelial dysfunction and vasoconstriction than low-risk patients, these systemic endothelial and vascular changes lead to suppression of angiogenesis or vascular permeability in the choroidal tissue.

Quantitative studies have reported conflicting results regarding CT changes in patients with severe preeclampsia. Iida et al. reported that choroidal circulation appeared to be more severely affected than the retinal circulation in preeclampsia patients, and that indocyanine green angiography disclosed hyperpermeability of choroidal vessels, which suggested severe damage to choroidal vascular walls [8]. Regarding vascular hyperpermeability, Evcimen et al. suggested that it is derived from a complex imbalance of choroid, involving increased hydrostatic pressure, choroidal ischemia, and endothelial cell dysfunction [27]. Garg et al. demonstrated subfoveal choroidal thickening in the setting of severe preeclampsia due to rising levels of VEGF [9], and Kim et al. reported that CT was increased in patients with preeclampsia and normal in healthy pregnant patients [28]. In contrast, Duru et al. found that subfoveal CT was significantly decreased in pregnant women with preeclampsia than in healthy pregnant women [29]. Atas et al. reported that using OCT, CT was increased both in women with preeclampsia and healthy pregnant women. Still, the increase in CT in preeclampsia was lower than that in healthy pregnant controls, which attributed to the markedly increased systemic vascular vasospasm secondary to preeclampsia [30]. Shim et al. suggested that there was a positive correlation between CT in preeclampsia and PCR in urine, implying preeclampsia severity [24]. This suggestion may be inconsistent with our findings, because it is a paradox that the changes of CT according to those of PCR and sFlt-1/PlGF ratio have opposite directions though both PCR and sFlt-1/PlGF ratio are indicators implying severe preeclampsia. However, considering that the CT is thickened as PlGF increases [16], our result is consistent with the effect of cytokines. In this sense, our result is inevitably conflicting, because sFlt-1/PlGF ratio is another biomarker of preeclampsia severity. As our study investigated the correlation between changes of the CT and the sFlt-1/PlGF ratio, we believe that these results may be attributed to the direct effect and functions of each cytokine. Our results revealed a direct association between the CT and cytokines, rather than the assumption that the serum markers and the CT may behave in identical directions depending on the severity of preeclampsia. Therefore, our results disclosed that these assumptions were doubtful and that the cytokines and ophthalmic parameters may react in a more complex way than shown by these simple assumptions.

Regarding the controversial CT results in preeclampsia, we speculate that the biomarkers may not be consistent in all preeclampsia due to the heterogeneous nature of the disease. Moreover, the CT in such cases may easily change according to the levels of various cytokines, which are changeable depending on the gestational age (GA) or preeclampsia phase. In addition, as the sFlt-1/PlGF ratio is a predictor of preeclampsia severity at 2 weeks after the current clinical status, the peak timings of CT, sFlt-1/PlGF ratio, and proteinuria levels may differ [31]. The hypothetical explanation of the conflicting results is as follows: high sFlt-1/PlGF ratio induces placental ischemia and the development of preeclampsia; it can be a predictor for the development of preeclampsia; high anti-angiogenic factor (sFlt-1) and low angiogenic factor (PlGF) induce vasoconstriction in the choroid and reduce choroidal vessel permeability, resulting in reduced CT; following that period, many cytokines may be released to compensate for the placental ischemia, which may increase vascular permeability, resulting in proteinuria or thick choroid, or offset the effect of the high sFlt-1/PlGF ratio.

A few studies have evaluated the microvascular structure of the retina using OCT-A in pregnant women. Saiko et al. reported that OCT-A noninvasively visualized ischemic changes in the choriocapillaris in hypertensive choroidopathy in pregnant women [32]. Ciloglu et al. evaluated the FAZ and VD of SCP and DCP using OCT-A in groups of pregnant women with preeclampsia, healthy pregnant women without preeclampsia, and healthy age-matched volunteers [33]. There was no significant difference in FAZ among the three groups; however, the VD of DCP and SCP was significantly lower in the preeclampsia group than in the healthy pregnancy group. They further revealed that DCP was more susceptible to generalized vasospasm due to its high metabolic activity and complex structure. In our study, there was no significant association between the retinal factors (FAZ, VD, and GCL+) in the OCT-A and the sFlt-1/PlGF ratios. These results are attributed to the relatively low vessel density of the inner and outer retina compared with the choroid tissue. Although systemic vasospasm and vasoconstriction affect retinal microvasculature, this effect may not be enough to anatomically change the microvascular structure, partially assisted by the hydrostatic pressure of vessels. However, as the choriocapillaris supplies the retina, long-term exposure to systemic vasospasm can alter the superficial and deep retinal vessels.

Serous retinal detachment (SRD) was observed in three of our patients. In one patient, SRD was identified 2 days after delivery; the sFlt-1/PlGF ratio was 283.17. Her SRD completely resolved 24 days after delivery, while the sFlt-1/PlGF ratio also improved to 95.69. In another patient, SRD was observed 4 days after delivery, and the sFlt-1/PlGF ratio was 1349.37. SRD resolved completely at 44 days after delivery, but her sFlt-1/PlGF ratio could not be measured due to an early discharge. The last patient who developed SRD prior to delivery underwent an emergency cesarean section operation, and SRD resolved 28 days post-operation. Her post-operative sFlt-1/PlGF ratio was 19.00. The sFlt-1/PlGF ratios of prior two patients were higher than the median value of 38. Although statistical analysis cannot be performed due to the small number of patients, we found that the sFlt-1/PlGF ratio decreased as SRD resolved in one patient, and a high sFlt-1/PlGF ratio seems to be associated with delayed recovery from SRD.

Our study had several limitations, including the small number of patients. Although the authors collected data of patients with preeclampsia in specific trimesters, GA varied and was not controlled. The choroid is sensitive to GA; hence, the CT may differ based on the GA.

In conclusion, CT was significantly reduced in preeclampsia with a high sFlt-1/PlGF ratio, and CT showed a negative association with the sFlt-1/PlGF ratio.
